# Supplementary material for: Severe Juvenile-Onset Systemic Lupus Erythematosus: A Case Series-Based Review and Update
Source: Children (Basel). 2023 May 10;10(5):852. doi: 10.3390/children10050852 (PMC10217445; doi:10.3390/children10050852)
Supplement: Supplementary file 1 [file children-10-00852-s001.zip › children-2360222-supplementary.pdf]

Supplementary materials

Table S1: EULAR diagnostic criteria for aSLE.

**All: ANA > 1:80**			*points*
**CLINICAL DOMAINS**	Constitutional	Fever	2
	Cutaneous	Non-scarring alopecia	2
		Oral Ulcers	2
		Subacute cutaneous or discoid lupus	4
		Acute cutaneous lupus	6
	Arthritis	Synovitis or tenderness in at least 2 joints	6
	Neurological	Delirium	2
		Psychosis	3
		Seizure	5
	Serositis	Pleural or pericardial effusion	5
		Acute pericarditis	6
	Hematologic	Leukopenia	3
		Thrombocytopenia	4
		Autoimmune hemolysis	4
	Renal	Proteinuria > 0,5 g/24h	4
		Class I or II lupus nephritis	8
		Class III or IV lupus nephritis	10
**IMMUNOLOGICAL DOMAINS**	Antiphospholipid antibody	Anti-cardiolipin IgG > 40 *or*<br>Anti-B2-glycoprotein 1 IgG > 40 *or*<br>Lupus anticoagulant	2
	Complement protein	Low C3 or low C4	3
		Low C3 and low C3	4
	High specific antibodies	Anti-dsDNA antibody *or*<br>Anti-SM antibody	6
